# Perceiving what you intend to do from what you do: evidence for embodiment in social interactions

**DOI:** 10.3402/snp.v5.28602

**Published:** 2015-08-04

**Authors:** Francois Quesque, Yann Coello

**Affiliations:** UMR CNRS 9193 SCALab, University of Lille, Lille, France

**Keywords:** perception, motor action, social intention, embodiment, kinematics

## Abstract

Although action and perception are central components of our interactions with the external world, the most recent experimental investigations also support their implications in the emotional, decision-making, and goal ascription processes in social context. In this article, we review the existing literature supporting this view and highlighting a link between reach-to-grasp motor actions and social communicative processes. First, we discuss the most recent experimental findings showing how the social context subtly influences the execution of object-oriented motor actions. Then, we show that the kinematic characteristics of object-oriented motor actions are modulated by the actor’s social intention. Finally, we demonstrate that naïve observers can implicitly take advantage of these kinematic effects for their own motor productions. Considered together, these data are compatible with the embodied cognition framework stating that cognition, and in our case social cognition, is grounded in knowledge associated with past sensory and motor experiences.

Compared to the vast majority of species in the animal kingdom, humans are very peculiar for the complexity of their social life (Wilson, 1975). In particular, human beings have developed the strong ability to adapt their behaviour as a function of the social context and to learn very quickly from observing conspecifics (Richerson & Boyd, 1998). Considering others’ behaviour purposely has furthermore resulted in the remarkable propensity to infer others’ intentions and mental states from their observable actions (Barresi & Moore, 1996). As a consequence, individuals engaged in social interactions tend to encode the behaviour of others in terms of their goal and meaning (Newtson, Engquist, & Bois, 1977; Vallacher & Wegner, 1987; Wegner & Vallacher, 1986), even when facing extremely rudimentary information as motion of abstract representation of social agents (Gergely, Nadasdy, Csibra, & Biro, 1995; Heider & Simmel, 1944; McAleer & Pollick, 2008; Rimé, Boulanger, & Laubin, 1985; Scholl & Tremoulet, 2000; Tremoulet & Feldman, 2000) or when reading text describing others’ actions (Hassin, Aarts, & Ferguson, 2005; Long & Golding, 1993; Poynor & Morris, 2003). Interestingly, goal ascription of observed actions occurs very early in life (Meltzoff, 1995; Meltzoff & Gopnik, 1993) and seems to happen without the need of explicit control of attention or conscious processing (Hassin, Aarts & Ferguson, 2005).

Furthermore, growing evidence suggests that people do not only infer the underlying goals of others actions through the observation of their motor behaviour, but they also tend to unconsciously adopt these goals and produce congruent actions (Brass, Bekkering, Wohlschläger, & Prinz, 2000; Chartrand & Bargh, 1999; Kilner, Paulignan, & Blakemore, 2003; Liepelt, Cramon, & Brass, 2008; Ondobaka, de Lange, Newman-Norlund, Wiemers, & Bekkering, 2011; Sebanz, Knoblich, & Prinz, 2003). In the field of social psychology, this well-known ‘goal contagion’ effect is thought to be highly adaptive by allowing individuals to appreciate the motivational reasons guiding others explicit behaviour and then anticipate their consequence and prepare to react (Aarts, Gollwitzer, & Hassin, 2004). Although yet debated (Goldman & de Vignemont, 2009), such spontaneous goal inference and activation of action tendencies in social situations may find their roots in unconscious embodied simulation processes (Gallese, 2003). According to the embodied framework, observed purposeful behaviour is interpreted and anticipated through simulation processes in the perceiver, which create a link between the observed action and the observer motor system. Supporting this view, recent works have suggested that evaluative responses can spontaneously emerge from embodied states, notably in a social context (Barsalou, 2003, 2008). These evaluations – driven by bottom-up processes – may automatically emerge from the activation of internal states linked to perception and elicit motor responses in relation to a specific intentional context. Our ability to predict the intention that drives another person’s action could then strongly rely on low-level mechanisms such as sensory-motor integration.

A fundamental distinction in our ability to read others’ mental states has however been made by Jacob and Jeannerod (2005). They stated that motor intentions (the intended effect of a goal-directed action in the environment) could be accurately inferred from the mere observation of voluntary motor actions. For instance, grasping a glass to bring it to a new position or to throw it away results in a different kinematic pattern of the reach-to-grasp action (Marteniuk, MacKenzie, Jeannerod, Athenes, & Dugas, 1987). By contrast, Jacob and Jeannerod (2005) suggested that social intentions (the intended effect of a goal-directed action on conspecifics) cannot be inferred from the mere observation of a voluntary motor action because different social intentions can be associated with the very same motor intention.^1^ For instance, grasping a glass of wine on the table at the end of a ceremony is thought to be independent of whether the intention is to drink the wine (individual intention) or to offer it to a friend waiting behind (social intention). Thus, the point emphasised by Jacob and Jeannerod was that motor intentions are identifiable from observed motor actions, but social intentions are obviously not.

However, recent data have challenged this view by showing that the social context also impacts on movement kinematics (for reviews see, Ansuini, Cavallo, Bertone, & Becchio, 2014; Becchio, Sartori, & Castiello, 2010). Ansuini et al. (2014) argued that by confronting internal predictions derived from the context of observed actions, it is in principal possible for a perceiver to identify social intentions from observed goal-directed motor actions. Their claim was that humans highlight specific kinematic signatures when intending to interact with conspecifics, which is thought to be one aspect of the communicative processes. Importantly, these spatio-temporal variations must be consistent to confer a benefit in multi-agent cooperative tasks. In support of this, we review in this paper the most recent findings showing that the very same action can be performed differently in function of the social context and the social intention endorsed by the actor, even when the motor intention is critically identical. In the first place, we will discuss experimental works showing how the social context subtly influences the execution of an object-oriented motor action. Then, we will show that the kinematic characteristics of an object-oriented motor action are modulated by the actor’s social intention. Finally, we will demonstrate that naïve observers can implicitly infer the social intention that drives motor action and take advantage from these kinematics effects for their own motor productions.

## Effect of the social context on goal-directed motor performances

During the last decades, numerous studies have investigated the role of social context on the planning and execution of a voluntary motor action. Initially, researchers have contrasted movements performed in the presence of a partner involved in the experimental task, to similar movements executed in isolation or in the presence of a passive observer (Becchio, Sartori, Bulgheroni, & Castiello, 2008b; Georgiou, Becchio, Glover, & Castiello, 2007; Quesque, Lewkowicz, Delevoye-Turrell, & Coello, 2013). A particular interest was also accorded to the characteristics of the partner and to the role of the relationship between the different agents participating in the experimental situation (Becchio, Sartori, Bulgheroni, & Castiello, 2008a; De Stefani, Innocenti, Secchi, Papa, & Gentilucci, 2013; Gianelli, Scorolli, & Borghi, 2013). Because a voluntary motor action is mainly determined by the target object’s characteristics and action goal, motor performances were thought to be independent of whether the motor task was performed in the presence or absence of other individuals, whatever their characteristics. Contrasting with this assumption, Quesque et al. (2013) found that the kinematic characteristics of a reach-to-grasp action were modulated by the relative position of a partner (see also, Becchio et al., 2008b; Gianelli et al., 2013). Precisely, the motor action was not influenced by the mere presence of a partner located far from the table but was influenced by the partner when she was located close enough to be able to intervene on the target object. In the latter situation, participants performed more fluent movements, with lower acceleration peaks and with longer reaction times. Interestingly, an effect of the social condition was also found on the action performed to position the target object before the main action (preparatory action, Quesque et al., 2013). This indicates that the social context influences all actions that are performed even when they are irrelevant according to the goal of the task.

Furthermore, Gianelli et al. (2013) demonstrated that life experience shared between individuals also influences movement kinematics in reach-to-grasp action. Precisely, reaching movements were performed more slowly in the presence of a friend than a recently met confederate. The attitude of the partner during the interaction was also found to influence the execution of the grasping action (Becchio et al., 2008a) as well as the type of gesture expressed by the partners’ even when no social interaction was expected (De Stefani et al., 2013; Ferri, Campione, Dalla Volta, Gianelli, & Gentilucci, 2011). For example, participants reacted faster when facing an actor performing a ‘stop’ gesture than a ‘give me in the hand’ gesture. These last results could be related to the communicative intention implicitly endorsed by participants (Sartori, Becchio, Bara, & Castiello, 2009), and may thus reflect a tendency in humans to spontaneously engage in a communication process when placed in a social interaction context.

## How does social intention shape our motor actions?

Among all the social factors thought to influence movement’s kinematics, social intention has received a particular attention in the field of motor behaviour. As mentioned above, social intention was defined by Jacob and Jeannerod (2005) as the ‘intention to affect a conspecific’s behaviour’ (pp. 22). According to these authors, different levels of intention are subordinate. Among them, motor intention – or intention in action – refers to the implementation of the execution of voluntary action, as for example displacing a glass at the centre of the table. However, more abstract private intentions can also be at the origin of this motor intention. For instance, a glass can be put at the centre of the table in order to increase the size of our close workspace, or in order to allow another person to reach it. In such situation, the spatial constraints of the task influence movement parameters, and this can be anticipated by the observer (Lewkowicz, Delevoye-Turrell, Bailly, Andry, & Gaussier, 2013; Marteniuk et al., 1987; Méary, Chary, Palluel-Germain, & Orliaguet, 2005). However, because this is the very same action that would be used to serve personal and social concerns, it was postulated that even if it is possible for an observer to detect motor intention from movement kinematics, she would be by no means able to detect social intention (Jacob & Jeannerod, 2005; de Vignemont & Haggard, 2008).

**Fig. 1:**
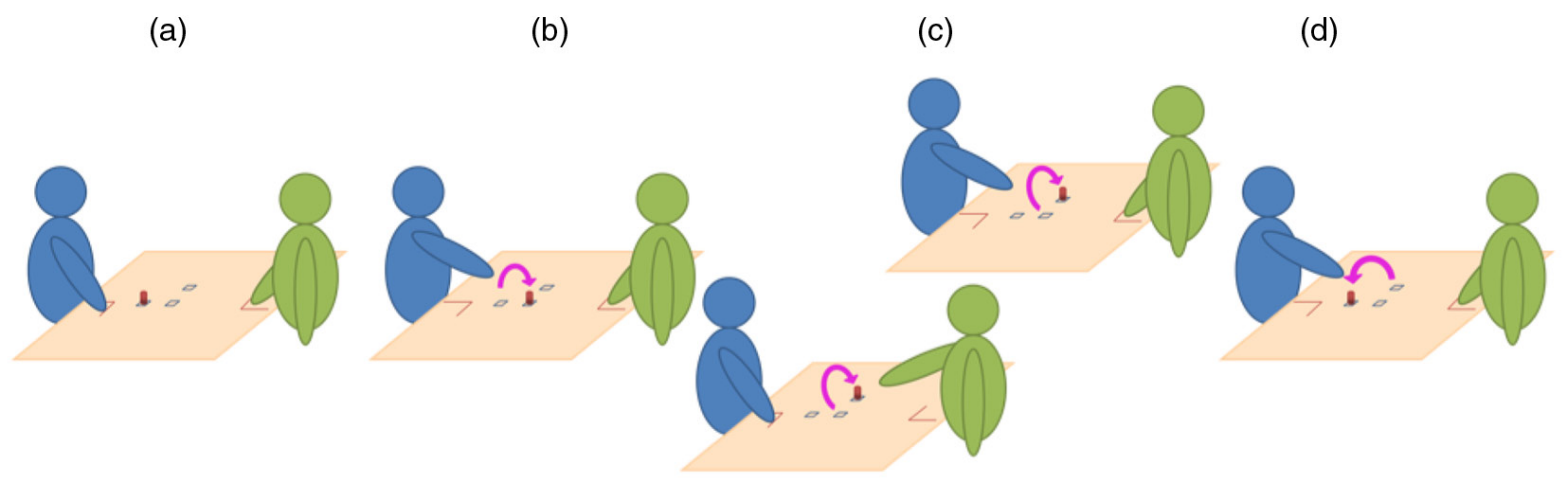
Representation of the actions’ sequence in the study of Quesque et al. (2013). The sequence always started with the wooden dowel placed on a nearby location and with the participant (in blue) and the partner (in green) pinching their index finger and thumb together on their respective starting positions (a). The Preparatory Action (b) consisted of displacing the wooden dowel from the nearby to the central location and was always performed by the participant, with no temporal constraint. The Main Action (c) consisted of displacing the wooden dowel from the central to the lateral location and could be performed either by the participant or by her partner, under strict temporal constraint. Finally, the Repositioning Action (d) was always performed by the participant and consisted of displacing the wooden dowel from the lateral to the nearby location, making the setup ready for the next trial.

Becchio et al. (2008b) were the first to experimentally investigate this issue. They requested participants to perform a reach-to-grasp action towards an egg-shaped object and to put it in a concave base (individual condition) or to put it in the opened hand of a partner seated at the table near to the participants (social condition). By comparing the kinematic profiles between these two conditions, they observed that when participants performed the reach-to-grasp movement in the social context, they tended to perform more curved trajectories and to produce actions with longer movement duration, compared to the individual condition. Though this might be viewed as an effect of social intention on motor performance, Jacob (2013) pointed out that the characteristics of a transitive action is known to be affected by the perceptual complexity of the landing site, leaving open the issue of the effect of social intention of motor performances. To investigate the effect of social intention more deeply, it was needed to modulate the social intention of a reach-to-grasp action while keeping unchanged the physical constraints of the task. This is explicitly what Quesque et al. (2013) tested, by comparing the effect of social intention in a sequential motor task. In their study, participants performed a preparatory action (consisting of displacing an object from a nearby to a central location) before performing a main action (consisting of displacing the object from the central to a lateral location). Only the main action was performed under temporal constraints (above 80% of the possible maximum speed, see Fig. 1). By informing the participant before the execution of the preparatory action about who will subsequently perform the main action, it was possible to impose the realisation of the same motor action towards the same physical target, but with different social intentions (placing the object at the central location for a subsequent personal use or for another person). Analysing kinematic profiles of the preparatory action, Quesque et al. (2013) observed that compared to the movements performed with a personal intention, movements performed with a social intention had longer durations, higher elevations, and longer reaction times, demonstrating that social intention modulates kinematics characteristics of a goal-directed action even when the physical constraints of the task are kept unchanged.

Temporal and kinematic variations observed in voluntary motor actions when participants endorse a social intention could be interpreted as a tendency in social context to implicitly provide informative signals to conspecifics about the current aim of a motor action (Sartori et al., 2009). In accordance with this view, Quesque et al. (2013) suggested that such exaggerations of the movement characteristics (slower actions and higher amplitudes) in interactive context could be implicitly implemented in order to attract the partner’s attention and give her time to prepare an adaptive motor response and cooperate appropriately. This interpretation is supported by the finding that humans tend to increase the amplitude of their movements when performing intentional communicative object-related actions compared to non-communicative object-related actions (Hermsdörfer, Hentze, & Goldenberg, 2006; Hermsdörfer, Li, Randerath, Goldenberg, & Johannsen, 2012). Moreover, it has been shown that when pointing an object to a partner with the arm, the pointing trajectories vary in relation to the relative location of conspecific (Cleret de Langavant et al., 2011). The exaggeration of the vertical amplitude of the grasping movement observed in socially-motivated actions could then reflect a specific allocation of attention to both the object to be grasped and the partner, the two constituting relevant sources of information in interaction context. In this respect, numerous studies have underlined the predominant role of gaze in social interactions (Argyle & Cook, 1976; Becchio, Bertone, & Castiello, 2008; Kleinke, 1986; Langton, Watt, & Bruce, 2000). It was also shown that not only the availability of the partner’s gaze influences individual behaviour (De Stefani et al., 2013; Ferri et al., 2011; Innocenti, De Stefani, Bernardi, Campione, & Gentilucci, 2013), but also gaze’s direction (Boucher et al., 2012), which is a reliable indicator of the intention to interact (Allison, Puce, & Mc Carthy, 2000; George & Conty, 2008; Senju & Johnson, 2009).

In this context, Quesque and Coello (2014) tested the role of a partner’s eye level on the social modulation of the trajectory curvature in a sequential motor task. An experimental paradigm similar to the one depicted in Fig. 1 was used, composed of a preparatory action always performed by the participant and a main action performed by either the participant or the partner (the experimenter in this study). In addition, the eye level of the partner was manipulated using an adjustable seat before the introduction of the participants to the experimental room. Participants came to the laboratory to perform the same task in two different sessions on different days (they were told that the researchers were interested in motor-learning abilities as a cover story). In one of the sessions, the partner was seated at the same height as the participant, whereas in the other session he was seated 5 cm higher. Results corroborated previous findings (Quesque et al., 2013) concerning the effect of endorsing a social intention, with an exaggeration of the temporal and spatial characteristics of the preparatory action. More interestingly, the spatial parameters of the preparatory action were found to be influenced by the relative eye level of the partner. The higher the partner eye level, the more the participants exaggerated the vertical curvature of their movements. These results confirm that a particular attention is allocated to human bodies when performing motor actions in a social context (Cleret de Langavant et al., 2012) and also supports that the presence of conspecifics automatically leads to considering their perspective (Mainwaring, Tversky, Ohgishi, & Schiano, 2003; Tversky & Martin Hard, 2009; Qureshi, Apperly, & Samson, 2010; Samson, Apperly, Braithwaite & Andrews, 2010) and to process objects in the environment with reference to them (Becchio, Bertone & Castiello, 2008). Finally, gaze direction which is known to be a highly valuable stimulus in social context, seems to induce in participants a particular attention resulting in a distortion of motor responses when they have the intention to socially interact, in accordance with other data (Chieffi et al., 2014). Considered together, these experiments support that the exaggerations of movement characteristics in a social interactive context are implemented in relation to the partner’s body properties. Because these alterations are not supposed to carry a benefit at the individual level, one may postulate that these variations are produced in order to facilitate partner’s detection of social goals of planned actions, and thus to enhance intuitive interactions between social agents.

## Are humans sensitive to socially-induced modulation of motor actions?

Being able to predict the actions of others represents a key ability for appropriate and efficient social interactions. Sport activity is the perfect illustration, as underlined by Hari and Kujala (2009). In football, for example, when willing to catch the ball the goalkeeper has to start moving before the ball is kicked and thus anticipate the goal of the player well before the entire execution of the action. Previous laboratory studies have shown that humans are very sensitive to kinematics variations of biological movements and are able to accurately – though often implicitly – anticipate a lot of information from movement observations. An object’s weight for instance can be evaluated through movement kinematics of a partner manipulating a (non-visible) object (Maguinness, Setti, Roudaia, & Kenny, 2013; Meulenbroek, Bosga, Hulstijn, & Miedl, 2007; Runeson & Frykholm, 1983). It is also possible to detect the deceptive intentions of a person performing an object-related action or even to have an idea of what weight the actor expected the object to be (Runeson & Frykholm, 1983). Moreover, when observing an action performed by someone else, it is also possible to detect the motor intention guiding that action from the very beginning of its execution (Lewkowicz et al., 2013; Méary, Chary, Palluel-Germain, & Orliaguet, 2005). In their study, Lewkowicz et al. (2013) presented short videos clips of object-directed arm reaching movements to naïve participants. Their task was to answer after each presentation whether the object in the video was reached by the actor to be placed either at the centre of the table, at the other side of the table, or close to them (the second part of the action was not shown). Results revealed that participants were able to anticipate the end-result of the grasping action from its early kinematic variations. Finally, recent works have shown that not only motor intention but also private mental states (Patel, Fleming, & Kilner, 2012) or even social intentions (Manera, Becchio, Cavallo, Sartori, & Castiello, 2011; Sartori, Becchio, & Castiello, 2011) can be perceived from observed motor performances. In these studies, the authors analysed participants’ ability to detect action intention in temporal-occlusion video tasks. Participants were asked to discriminate between reach-to-grasp movements performed at fast or slow speed and reach-to-grasp movement performed with the intention to cooperate or to compete with a partner. Participants were able to correctly categorise the observed motor action performed with different social intentions, and interestingly, their performances were not altered by the presentation of point-light display versions of the videos stimuli (Manera et al., 2011), confirming thus that their perception was essentially based on kinematic information. A recent work led by Lewkowicz, Quesque, Coello and Delevoye-Turrell (In press) corroborates these conclusions. The authors asked their participants to explicitly categorise short video clips of actors performing a sequential motor task while endorsing social or personal intentions. The sequential task was that used by Quesque et al. (2013), consisting of a preparatory and a main grasping action (see Fig. 1). Only the preparatory action was shown in the videos. Furthermore, only the arm of the actors was visible in order to avoid any effect associated with posture or gaze variations (Sartori et al., 2011). In two distinct experiments, the authors observed that participants were able to correctly classify the stimuli in function of the social/personal intention of the actor. Moreover, to assess whether kinematic variations in the videos clips were determinant in the detection of social intention, video clips were normalised to control for variations of reaction time and movement time. Results showed that the detection of social intention relies on the integration of these kinematic parameters that are implicitly perceived in the grasping action. However, as underlined by Obhi (2012), in these experiments the choice set of possible intentions to be discriminate is experimentally constrained. It has been shown that humans can categorise social and non-social motor actions (Manera et al., 2011; Sartori et al., 2011), but this does not precisely validate that they implicitly detect social intention from movement kinematics. It may then be possible that an observer explicitly distinguishes movements driven by different intentions without the necessity to perceive what precise intention supports these actions and to use it in cooperative tasks. Whether humans can take advantage of the kinematics variations induced by a social interaction context for their own action, which would be of particular relevance in most of the social contexts, remains then, an issue that needs to be properly addressed.

**Fig. 2:**
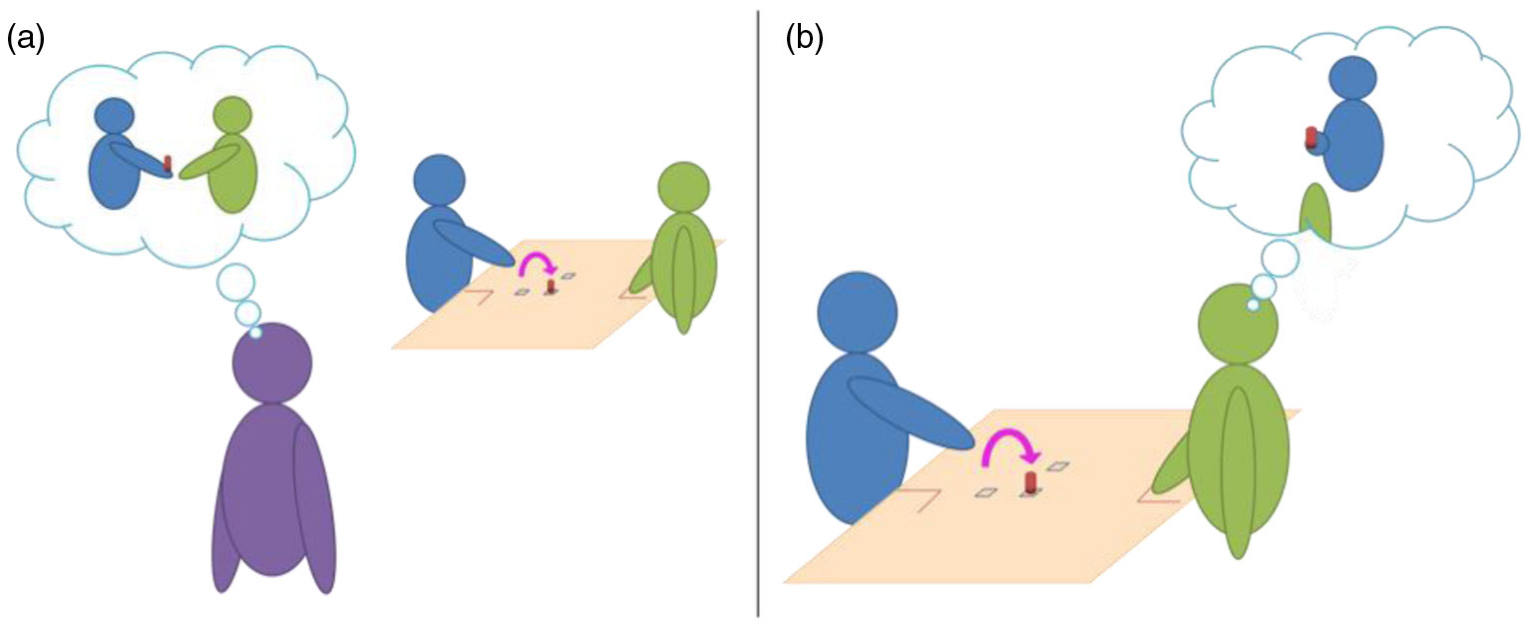
Illustrations of (a) the ‘third-person’ and (b) the ‘second-person’ perspective. Classical experimental paradigms built to investigate humans’ mind-reading abilities use a third-person perspective (through photos, videos, or point-light display presentation of an actor). If participants are able to correctly categorise the stimuli above the level of chance, nothing is said about their understanding of the underlying intention of the actor. Switching from a ‘third person’ to a ‘second person’ perspective would allow distinguishing between categorisation and mind-reading abilities. If social intentions can actually be grasped through the observation of movement kinematics in a cooperative task, participants’ behaviours should be influenced (facilitation or interference effect) in consequence.

In this respect, Manera, Del Giudice, Bara, Verfaillie, and Becchio (2011) showed that the perception of a movement performed with a communicative intention could prepare the perceiver for being involved in social interaction. In particular, when facing point-light displays of two moving agents, the perception of the second agent is facilitated when the first one performed a communicative gesture, in comparison to a control condition comprising non-communicative gesture. Thus, the information extracted from a communicative gesture influenced the processing of biological motion, showing moreover that facilitation effects can inform about the processing of social intention. Furthermore, a switch from the classical ‘third-person perspective’ to a ‘second-person perspective’ (see Fig. 2) has recently been pointed to as a clear necessity in the field of mind-reading studies (Ansuini et al., 2014; Schilbach, 2010).

In line with this approach, Quesque, Delevoye-Turell, and Coello (Under review) conducted an experiment to evaluate whether observers are implicitly sensitive to social intention in a cooperative task and whether this influences the planning of their own motor actions. In their study, the authors adapted the sequential motor task developed by Quesque et al. (2013) composed of a preparatory and a main action and tested dyads of naive participants. To control for the execution of the motor sequence, auditory cues were provided through headphones to an actor and a partner seated at a table and facing each other. Depending on the cue, either the actor or the partner had to perform the main action (i.e. displace a wooden dowel from a central to a lateral location as fast as possible). As this was the case in previous studies, before performing the main action the actor had to perform a preparatory action consisting of moving the wooden dowel from a nearby to the central location in response to a first auditory cue. This first cue could inform the actor about who will make the upcoming main action (the actor herself: ‘moi’ –myself, or the partner: ‘lui’-the other; 50% of the random trials) or could be non-informative (‘prêt’-ready; 50% of the random trials). The partner always received non-informative cues (‘prêt’-ready; 100% of the random trials). Confirming previous reports, the authors found that actors took more time to initiate their preparatory action and executed the reach-to-grasp movement with greater amplitude when placing the object for their partner (Quesque & Coello, 2014). The most striking finding, however, was that the partners showed a facilitation effect when performing the main action after the actors executed the preparatory action driven by a social intention (‘lui’-the other condition) compared to when performing it after the actor executed the preparatory action driven by a non-social intention, and despite the partners receiving consistently neutral information (‘prêt’-ready). Then, these results revealed that the partners not only produced different motor responses depending on perceived kinematic patterns, but that they were also able to take advantage of those movement signatures so as to produce more efficient main actions. This indicates that the detection of subtle kinematic variations in a social context could prime the perceiver to prepare for social interaction and anticipate appropriate motor responses. Finally, it is worth noting that all participants remained unaware of these effects, which supports the idea that the perception of social intention from action kinematics relies on low-level mechanisms and does not necessarily involve conscious inferences processes (Gallagher, 2008).

## Grasping social intention from social interactions

On the basis of the experimental evidences detailed above, it can be postulated that the understanding of others’ social intention is linked to our own motor system. Namely, this is because I am (or not) induced to perform a certain behaviour that I can spontaneously figure out the social scope of my partner’s motor action. In agreement with this framework, it has been shown that motor brain areas broadly contribute to perceptual predictions from observed motor actions and that action understanding and action preparation are supported by common processes (Chaminade, Meary, Orliaguet, & Decety, 2001; Filimon, Nelson, Hagler, & Sereno, 2007; Newman-Norlund, van Schie, van Zuijlen, & Bekkering, 2007). Through everyday experiences, situated conceptualisations grounded in perceptual and motor systems are stored in memory (Barsalou, 2008) and as a result of the repeated associations between actions and their effects, the mere perception of a given action can lead to automatic pattern completion from which emerges the meaning (Barsalou, 2013; Paulus, 2011). At the behavioural level for example, predictive eyes movements studies have revealed that humans can anticipate and look at the end of a motor action with a high accuracy, long before the action was entirely executed (Ambrosini, Costantini, & Sinigaglia, 2011; Elsner, Falck-Ytter, & Gredebäck, 2012; Flanagan & Johansson, 2003; Rotman, 2006). Furthermore, the visual perception of an identical action could activate specifically motor representations depending of the temporal and contextual characteristics of the situation (González-Perilli & Ellis, 2015), requiring or not a complementary collaborative action from the perceiver (Sartori, Bucchioni, & Castiello, 2013; Sartori, Cavallo, Bucchioni, & Castiello, 2011, 2012). The direct perception of the motor actions of another person could then drive a behavioural response and elicit related internal states, allowing the perceiver to subsequently understand the intentions behind these actions. Such a mechanism can easily explain the fact that humans adapt very quickly and involuntary their reactions to the actual private intention of a partner (e.g. to compete), even if they were explicitly informed that the partner follows an opposite goal (e.g. to cooperate, Becchio et al., 2008a). It seems then that the implicit processing of social variations embedded in motor features contributes to the activation of adapted responses and spontaneously informs the perceiver about the social demand. It is, however, interesting to note that this process could be modulated in function of the social characteristics (e.g. group membership, Gallagher & Varga, 2014) and particularly in function of the level of intimacy with the other person (Fitzsimons & Bargh, 2003; Shah, 2003) as suggested by the literature concerning the phenomenon of ‘goal contagion’.

## Conclusion

As previously mentioned, Jacob and Jeannerod (2005) postulated that observing the movement performed by someone else allows the observer to represent the actor’s motor intention but will not allow her to access to the actor’s social intention, because different social intentions can be associated with the very same motor intention. The studies discussed in this article challenge this view by showing that the execution of a same motor intention subtly varies in function with the social context in which it is performed. In particular, it has been detailed here that the kinematic characteristics of an object-oriented motor action are modulated by the proximity of conspecifics, and exaggerated when the actor endorse a social intention. Besides, we reported experimental evidences suggesting that other bodies’ characteristics are implicitly taken into account when we behave in a social context, probably to include in the motor productions implicit communicative information. Finally, we mentioned the last experimental evidence concerning this issue and showed that naïve observers do not only perceive these informative social cues but are also able to implicitly take advantage of them for their own motor performances. Considered together, our ability to predict others’ actions and ascribe intention and mental states to others seems to be highly grounded in the interactions between our body and the social environment. It is, however, important to note that if it is possible to perceive others’ private goals from their own actions, there is no experimental evidence yet showing that it would be possible to access to their beliefs. Apperly and Butterfill (2009) proposed a two systems account of theory of mind. A first inflexible system, which would be involved in fast and efficient attributions in response to others behaviours, and a second slower system, which would support more abstract and deliberate inferences. The literature reviewed so far strongly supports that the first inflexible system depends on embodied processing. Whether this is also the case for the second slower system remains an open question for future research.

## Footnotes

1 This refers to the well-known Dr. Jekyll and Mr. Hyde paradox, as described by Jacob and Jeannerod (2005). In the novella written by Robert Louis Stevenson, Dr. Jekyll, alias Mr. Hyde, is a renowned surgeon who performs appendectomies on his anesthetised patients, to heal them during the day but to murder them during the night. He then executes the same motor action during the day and at night, whereby he grasps his scalpel and applies it to the same bodily part of two different persons. According to Jacob and Jeannerod, Dr. Jekyll’s motor intention is the same as Mr. Hyde’s, although Dr. Jekyll’s social intention (treating patients) clearly differs from Mr. Hyde’s social intention (murdering victims). Social intention was thus thought to be hardly identifiable from movement characteristics.
